# Use of time-lapse technology and artificial intelligence in the embryology laboratory: an updated review

**DOI:** 10.5935/1518-0557.20250019

**Published:** 2025

**Authors:** Romualdo Sciorio, Luca Tramontano, Giuseppe Gullo

**Affiliations:** 1 Fertility Medicine and Gynaecological Endocrinology Unit, Department Woman Mother Child, Lausanne University Hospital, 1011 Lausanne, Switzerland; 2 Département de Gynécologie-Obstétrique, Réseau Hospitalier Neuchâtelois, 2000 Neuchâtel, Switzerland; 3 Department of Obstetrics and Gynaecology, Villa Sofia Cervello Hospital, University of Palermo, Italy

**Keywords:** time-lapse monitoring, medically assisted reproduction, embryo culture and selection, embryo evaluation, artificial intelligence, machine learning, ploidy prediction

## Abstract

During human *in-vitro* culture, morphological microscope analysis is routinely used to select embryos with the highest implantation potential for transfer, aiming for successful pregnancy and healthy live birth. This evaluation includes blastomere number, size, fragmentation, multinucleation, blastocyst (BL) expansion, and the inner-cell mass and trophectoderm appearance. However, this method requires removing embryos from the incubator, exposing them to non-physiological conditions such as fluctuations in pH, temperature, gases concentrations, as well as significant inter-observer variability. Continuous embryo culture using time-lapse monitoring (TLM) has revolutionized embryo evaluation by allowing continuous, real-time tracking of embryo development from fertilisation to blastocyst formation. This reduces the need to remove embryos from the incubator and helps maintain stable culture conditions. The monitoring system typically includes a standard incubator with an integrated microscope coupled to a digital camera, capturing images at regular intervals that are processed into a video for analysis. Despite its advantages, accurately predicting implantation rates in humans remains challenging. Recently, artificial intelligence (AI) has emerged as promising tool to objectively evaluate human embryos. AI can analyse large datasets, including embryological, clinical, and genetic information, and assist in individualizing treatment protocols. Integrating AI with TLM could improve embryo selection and enhance overall success rates. This paper explores the potential benefits of combining TLM and AI in reproductive and embryology laboratories, highlighting their potential to improve the outcomes of human ART.

## INTRODUCTION

Assisted reproductive technologies (ART) has advanced significantly over the past four decades, marked by several key milestones and historical achievements (Steptoe & Edwards, 1978). Since the birth of the first *in-vitro* fertilization (IVF) baby in 1978, over 10 million children have been born through IVF (European IVF Monitoring Consortium (EIM) for the European Society of Human Reproduction and Embryology (ESHRE), 2023). In recent years, the number of couples facing infertility has steadily increased, many of whom will eventually require IVF treatments. Worldwide, approximately 2.5 million ART cycles are performed each year, leading to over 500,000 births annually. In the UK, IVF babies account for about 3% of all babies born in 2016 (De Geyter *et al.*, 2018; Human Fertilisation & Embryology Authority, 2018).

Despite these advancements, *in-vitro* embryo development remains suboptimal, and many high-quality embryos fail to implant, resulting in no pregnancy (Zhao *et al.*, 2011; Niederberger *et al.*, 2018; European IVF Monitoring Consortium (EIM) for the European Society of Human Reproduction and Embryology (ESHRE), 2023). The development of more complex culture media has improved embryo quality, allowing for embryo transfer at the blastocyst (BL) stage. Extending embryo culture enables the selection of embryos at a more advanced stage, and improves both uterine and embryonic synchronicity, and thus enhances pregnancy success rates (Gardner & Schoolcraft, 1999; De Vos *et al.*, 2016; Gullo *et al.*, 2022). Furthermore, transferring a single BL minimizes the medical risks associated with multiple pregnancies for both the mother and the baby (Sullivan *et al.*, 2012).

However, the ability of embryologists to select the best embryo for transfer has not significantly changed since the birth of Louise Brown (Steptoe & Edwards, 1978). From the onset of IVF, it was recognized that the grade of embryo development was correlated with successful pregnancy (Edwards *et al.*, 1984). Traditionally, embryos are selected for transfer based on morphological evaluation, which provides a snapshot of their development. For cleavage stage embryos, factors such as the number and size of blastomeres, fragmentation, and multinucleation are examined (ESHRE Guideline Group on Good Practice in IVF Labs, 2016; Gullo *et al.*, 2023). For blastocyst assessment, the most widely used grading system was originally proposed by Gardner & Schoolcraft (1999). This alphanumeric system, while not covering all aspects of BL morphology, effectively classifies the appearance and compactness of the inner cell mass (ICM), the cohesiveness of the trophectoderm (TE) cells, and the degree of expansion of the blastocoels cavity (Gardner & Schoolcraft, 1999). However, morphological assessment has limitations in predicting implantation potential due to significant inter-observer variability (Braude, 2013).

Embryo development is a dynamic process, with morphology changing rapidly over short periods (Lemmen *et al.*, 2008). Although TLM was first reported by Lewis & Gregory (1929), and Payne *et al.* (1997), it has only recently been introduced into embryology laboratory. This technology allows scientists to analyse the entire sequence of embryo development. A time-lapse system comprises three key components: an incubator, a microscope, and software. Together, these elements provide continuous monitoring while maintaining a stable and uninterrupted culture environment, which avoids the need to move embryos out of the incubator (Meseguer *et al.*, 2012; Basile *et al.*, 2014; Aparicio-Ruiz *et al.*, 2016), preventing exposure to non-physiological conditions such as fluctuating temperature, humidity, pH and gas concentrations (Zhang *et al.*, 2010). Wong *et al.* (2010) found that the development of human embryos to the BL stage was linked to key timing events during early divisions, such as the duration of the first cleavage and the interval between the second and third divisions. In 2011, Meseguer *et al.* (2011) introduced the “morphokinetics” to describe the timing of cell division events, showing that embryo implantation was associated with specific cell timing parameters.

This review paper does not aim to provide scientific evidence of the TLM, as this has been recently investigated by Armstrong *et al.* (2018). Instead, the primary goal is to illustrate the various TLM systems available, discuss their potential benefits in the embryology laboratory, and advise IVF clinics on selecting the most suitable system based on their specific conditions.

## APPLICATION OF TIME-LAPSE TECHNOLOGY IN ART

Morphology has been the primary method of embryo assessment for over 40 years and remains the main approach for embryo selection during ART cycles. However, standard evaluations at specific time points have limitations, primarily due to the subjectivity of the embryologist and the inability to capture critical events that may affect embryo viability. Morphological assessment provides only a snapshot of embryo development at a specific moment, missing what occurs during the intervals between observations (Cruz *et al.*, 2012). Additionally, the embryo’s grade can change significantly over a short period (Wong *et al.*, 2010; Meseguer *et al.*, 2011). In contrast, the TLM not only allows for the assessment of embryo morphology and dynamic changes during the *in-vitro* development but also provides stable culture conditions (Wong *et al.*, 2010; Meseguer *et al.*, 2012; Basile *et al.*, 2014; Aparicio-Ruiz *et al.*, 2016).

Although pioneering research on TLM began in the late 1990s (Payne *et al.*, 1997), the technology became commercially available to embryology laboratories in 2009. The growing body of published articles on TLM in human embryology suggests its active application in embryology laboratories worldwide. However, there is limited data on its global use. Scotland is unique in this regard, as the government provided funding for all public assisted conception units (National Health Service - Scotland) to invest in TLM. Apart from this case, there are few studies reporting the worldwide use and implementation of TLM in ART cycles. One such study, by Dolinko *et al.* (2017), surveyed 294 IVF units in the USA. Of the 162 responding units, 35 laboratories reported using at least one time-lapse system. A similar report by Boueilh *et al.* (2018) from France found that among 78 respondents, 30 centres were using TLM clinically. While these surveys provide valuable insights into TLM use in two different countries, they are insufficient to draw conclusions about its global adoption. A comprehensive global survey would be valuable to assess the current use of time-lapse technology in IVF practices.

## TIME-LAPSE MONITORING AND CONTINUOUS EMBRYO ASSESSMENT FROM FERTILIZATION TO BLASTOCYST FORMATION

The identification of the embryos with the highest implantation potential and viability, leading to successful pregnancies, remains a challenging goal in ART cycles. This paragraph investigates whether the use of the TLM and morphokinetic embryo assessment can aid in achieving this goal.

Time-lapse observations have been used in defining new or poorly understood aspects of human embryology, such as the fertilization process (Coticchio *et al.*, 2018), the duration of the first three cell cycles (Wong *et al.*, 2010; Meseguer *et al.*, 2011), early compaction (Iwata *et al.*, 2014) and BL formation (Marcos *et al.*, 2015; Sciorio *et al.*, 2020a; 2020b). Coticchio *et al.* (2018) conducted an in-depth analysis of the fertilization event, identifying previously unknown characteristics, including the cytoplasmic halo (its appearance and disappearance), the fading of the pronuclei (PN), the time from PN fading (tPNf), and the first cleavage. These new features were used to predict embryo quality on day-3 (Coticchio *et al.*, 2018). A subsequent prospective study analysed the correlation between tPNf and live birth outcomes in 159 embryos. The pronuclei morphology of 46 embryos resulting in live birth was compared with that of 113 embryos that did not lead to live births. The results indicated that tPNf occurred significantly later in embryos that resulted in live births, never occurring earlier than 20 hours and 45 minutes (Azzarello *et al.*, 2012). Further study found that erratic PN movement and delayed PN fading were indicative of compromised embryo development (Athayde Wirka *et al.*, 2014). In a retrospective multicentre trial, the authors identified four atypical phenotypes, including abnormal syngamy, abnormal first cytokinesis, abnormal cleavage, and chaotic cleavage, which were correlated with embryo viability and implantation potential. The study concluded that embryos exhibiting these atypical phenotypes had significantly lower developmental potential compared to the control group (Athayde Wirka *et al.*, 2014). Wong *et al.* (2010) suggested that the BL stage could be predicted with high sensitivity based on the timing of early developmental events, including the first cytokinesis (0-33 minutes), the time interval between the end of the first mitosis and the initiation of the second (duration of the two-cell stage: 7.8-14.3 hours), and the time interval between the second and third mitoses (duration of the three-cell stage: 0-5.8 hours). Lemmen *et al.* (2008) found that embryos resulting in successful pregnancies exhibited significantly higher cleavage synchrony and better synchrony in nuclear appearance at the two-cell stage compared to non-implanting embryos. Using morphokinetics assessment, associations have been demonstrated between various cleavage stage events and an embryo’s ability to reach the blastocyst stage (Wong *et al.*, 2010; Cruz *et al.*, 2012). Meseguer *et al.* (2011) analysed large datasets from embryos generated by ICSI and found that the timing of cleavage to five cells was the most predictive parameters for embryo viability and implantation. In a retrospective multicentre study conducted across ten IVF clinics, they compared pregnancy outcomes between embryos cultured using TLM (n=1,390 cycles) and standard incubators (n=5,915). The study reported a 20% improvement in pregnancy rates in the TLM group, which was attributed to a combination of both stable culture conditions and the use of morphokinetic parameters for embryo selection (Meseguer *et al.*, 2012). Similar results were confirmed in a prospective *randomized controlled trial* conducted by the same group two years later (Rubio *et al.*, 2014).

The introduction of more physiological culture conditions for *in-vitro* human embryos has led to the routine culture and transfer of embryos at the BL stage (Gardner & Schoolcraft, 1999; De Vos *et al.*, 2016). In countries following a policy of single embryo transfer, there has been a notable reduction in the number of embryos being transferred. Additionally, the transfer of a single BL prevents the adverse medical conditions associated with multiple pregnancies (Sullivan *et al.*, 2012; De Vos *et al.*, 2016). Furthermore, blastocyst transfers result in higher implantation rates compared to transfers at the cleavage stage, although this outcome needs to be considered alongside potential detrimental epigenetic effects associated with extended *in vitro* culture (Kirkegaard *et al.*, 2012). In this context, TLM has been used to predict BL formation and implantation potential based on novel morphokinetic parameters observed during the cleavage stage (Dal Canto *et al.*, 2012). Kirkegaard *et al.* (2013) reported that cleavage from the two-to eight-cell stage occurs progressively earlier in embryos that will form a BL and successfully implant. They suggested high-quality blastocysts could be predicted within the first two days of *in-vitro* culture based on a short duration of the first cleavage and the duration of the three-cell stage (Kirkegaard *et al.*, 2013). Similarly, Hashimoto *et al.* (2012) showed that higher-quality blastocysts exhibited significantly shorter times for synchrony between the three and four cell stages. More recently, Motato *et al.* (2016) assessed the morphokinetic parameters in 7,483 embryos and identified two features linked to BL formation: the time of morula formation (81.28-96.0 hours after ICSI) and the transition from five to eight cell embryos (≤8.78 hours). Finally, spontaneous BL collapse during *in*-*vitro* embryo development has been suggested as a novel marker of embryo viability and implantation potential. Retrospective studies have shown that BL exhibiting collapse during development are less likely to implant and result in a pregnancy compared to those that do not (Marcos *et al.*, 2015; Sciorio *et al.*, 2020a; 2020b). Annotation of collapse events may improve embryo assessment at blastocyst stage.

A summary of the main atypical features identified with the TLM and some published papers are presented in Tables 1 and 2.

**Table 1:** Atypical features that can be identified with time-lapse monitoring incubator.

Feature	Description	Study/Reference
-Pronuclei (PN) formation -Singamy	Wrong PN movement in the cytoplasm	Coticchio *et al.*, 2018 Azzarello *et al.*, 2012
-Appearance of two PN -Pronuclei reappearance	-Asynchronous appearance and disappearance of PN -Pronuclei fading and reappearance	Coticchio *et al.*, 2018
-Pronuclei size	Difference in pronuclear areas before pronuclear fading	Otsuki *et al.*, 2017
-PN fragmentation -PN fusion	-Formation of micronuclei -A pronucleus formed by the fusion of two preexisting pronuclei	Mio & Maeda, 2008 Coticchio *et al.*, 2018
- Unipolar cleavage furrow -Tripolar cleavage furrow -Pseudofurrows	-Appearance of cleavage furrow on one site of the zygote -Appearance of three cleavage furrows -Zygote presenting oolemma ruffling before cytokinesis	Wong *et al.*, 2010 Athayde Wirka *et al.*, 2014
-Absent cleavage -Reverse cleavage	-Arrest at zygote stage -Fusion of two cells into one blastomere	Barrie *et al.*, 2017 Desai *et al.*, 2014
Direct cleavage	Cleavage of zygote to three cells or one blastomere divides to three cells	Athayde Wirka *et al.*, 2014 Barrie *et al.*, 2017 Meseguer *et al.*, 2011
Blastomere movement	Blastomere and cytoplasm movement before division	Ezoe *et al.*, 2019
Multinucleation	Blastomere with more than one nucleus	Desai *et al.*, 2014 Hashimoto *et al.*, 2016
Internalization of cellular fragments	Fragments reabsorbed into one blastomere	Mio & Maeda, 2008
Irregular chaotic division	Disordered cleavage behaviour with uneven cleavages and fragmentation	Athayde Wirka *et al.*, 2014 Barrie *et al.*, 2017 Meseguer *et al.*, 2011
Early compaction	Formation of tight junctions between blastomeres in day 3 embryos	Iwata *et al.*, 2014
Cell exclusion	Exclusion of one or more blastomeres from the morula formation	Coticchio *et al.*, 2019)
Spontaneous Blastocyst collapse	Collapse of blastocyst with complete disappearance of blastocoel cavity	Marcos *et al.*, 2015 Sciorio *et al.*, 2020a Sciorio *et al.*, 2020b

**Table 2:** Some manuscripts published from 2010 that have used the time-lapse technology.

Study	Aim/Description
Azzarello *et al.*, 2012	Pronuclei (PN) development in embryos after ICSI
(Athayde Wirka *et al.*, 2014)	Identification of atypical embryo phenotypes by time-lapse, and correlation with embryo development
Aparicio-Ruiz *et al.*, 2016	To correlate morphokinetic parameters with blastocyst formation, quality, implantation and ongoing pregnancy rates
Aguilar *et al.*, 2014	Time correlation between fertilization events and embryo implantation
Armstrong *et al.*, 2018	Time-lapse cochrane review
Basile *et al.*, 2014	Elaborate with use of time-lapse an algorithm to increase the probability of noninvasively selecting a chromosomally normal embryo
Boueilh *et al.*, 2018	Evaluation of time-lapse imaging used in French IVF units
Coticchio *et al.*, 2018	Time-lapse analysis and novel aspects of human fertilization and new morphokinetic parameters of embryo viability
Cruz *et al.*, 2012	Correlation between embryo division kinetics and blastocyst formation
Chavez *et al.*, 2012	Precise cell cycle parameter timing is observed in all euploid embryos to the four-cell stage, whereas only 30% of aneuploid embryos exhibit parameter values within normal timing windows
Campbell *et al.*, 2013a	Develop a model to categorize the risk of embryo aneuploidy based on morphokinetic parameters
Campbell *et al.*, 2013b	Evaluate the effectiveness of the previously established, morphokinetic-based aneuploidy risk classification model
Chawla *et al.*, 2015	To analyse differences in morphokinetic parameters of euploid and aneuploid embryos utilizing time-lapse imaging and genetic analysis
Chen *et al.*, 2013	Time-lapse review
Dal Canto *et al.*, 2012	Analyse cleavage timings in relation to blastocyst formation and implantation
Fréour *et al.*, 2013	Evaluate of embryo morphokinetic parameters and female smoking status
Hashimoto *et al.*, 2012	Assess the development kinetics of embryos and their ability to develop to blastocyst
Iwata *et al.*, 2014	Analyse the timing of initiation of compaction in human embryos
Kirkegaard *et al.*, 2013	Duration of the first cytokinesis, duration of the 3-cell stage and direct cleavage to 3-cells predicted development to high-quality blastocyst
Meseguer *et al.*, 2011	Generate and evaluate an embryo selection tool based on morphokinetics
Meseguer *et al.*, 2012	Compare pregnancy outcomes in an incubator with time-lapse versus tissue culture chamber
Motato *et al.*, 2016	To correlate morphokinetic parameters with blastocyst formation and implantation
Muñoz *et al.*, 2013	Evaluate if type of GnRH analog used during controlled ovarian stimulation influences early embryo developmental kinetics
Montag, 2013	Time-lapse review attempts to correlate timings with embryonic aneuploidy
Racowsky *et al.*, 2015	Time-lapse review
Rubio *et al.*, 2012	Analyse implantation rate of embryos with cleavage from 2 to 3 cells in less than five hours
Sciorio *et al.*, 2020a, 2020b Marcos *et al.*, 2015	Time-lapse imaging showed that blastocyst collapse(s) event is associated to low implantation potential
Sundvall *et al.*, 2013	To assess the variability of time-lapse annotations
Swain, 2013	Review of studies that attempt to correlate timings with embryo aneuploidy
Wong *et al.*, 2010	Prediction of embryo potential to blastocyst stage using morphokinetic parameters

## DIFFERENT TLM SYSTEMS

At present, several commercially available time-lapse systems are used in reproductive laboratories for embryo selection. When selecting a TLM model, the clinic should consider various practical aspects, including the size and space requirements of each system, the cost, and the laboratory workload. In general, all systems require the use of a digital inverted microscope coupled with a camera to capture embryo images at specific time intervals. Some models feature an incubator that is equipped with a built-in camera, while other systems utilize a camera placed inside a traditional large-box incubator (Kirkegaard *et al.*, 2012; Chen *et al.*, 2013; Campbell & Fishel, 2015).

While all currently available TLM systems use an oil overlay on culture microdrops to prevent evaporation, there are differences in how embryos are cultured, and each system requires a specific culture dish supplied by the manufacturer. Some models offer individual culture set-up, where each dish has a designed number of wells, with each well holding one embryo (Chen *et al.*, 2013; Campbell & Fishel, 2015; Racowsky *et al.*, 2015). In contrast, other culture dishes allow for the sharing of culture media between compartments, making them suitable for group culture, which enables exchange of soluble components between embryos. This design may have implications for embryo development and could be an important consideration when selecting a specific model to purchase.

Additionally, each system uses a different light source and differs in the method used to bring embryos into the field of view. Some systems do not move the embryos during imaging, while others involve constant movement of the culture dish. The choice of light source also impacts the type of imaging technology used. A few systems use bright-field technology, which allows for the assessment of both kinetic parameters and embryo morphology. Other models use dark-field technology, which supports the determination of kinetic parameters but provides limited information on the morphological features of the embryos.

Other factors influencing the selection of a TLM system include the nature of the computer software used for visualisation and analysis, as well as the options for annotation. Annotations can be added either manually or automatically, which may affect the efficiency and accuracy of embryo assessment.

Finally, the selection of a time-lapse system is a critical decision that involves considering numerous factors such as system configuration, imaging technology, and software features to ensure optimal embryo evaluation and selection.

## POTENTIAL BENEFIT OF TLM AND ITS IMPACT ON EMBRYO CULTURE

Human embryo culture involves various physical and chemical stressors (Wale & Gardner, 2016), which can create a hostile environment for the pre-implantation developing embryo. TLM provides consistent culture conditions, minimizing exposure to non-physiologic values, and enabling more reliable observation and assessment of the embryo development, thus improving the chances of a successful pregnancy. The culture media used to grow human embryos is another critical factor influencing embryo development. Over the past few decades, considerable improvements have been made in the composition of culture media, aimed at optimizing the conditions for embryonic growth. Two main approaches have been suggested: sequential media and single-step media.

Sequential media are designed to mimic the in vivo environment by providing different chemical compounds to the developing embryo, similar to its natural transition from the oviduct to the uterus. This media is typically tailored to meet the changing needs of the embryo as it progresses through different stages of development (Barnes *et al.*, 1995). In contrast, the single step media is based on the concept that providing all the necessary metabolic nutrients from the outset allows the embryo to utilize these nutrients as needed, according on its specific developmental demand (Summers *et al.*, 1995). While both methods have been widely used, the debate over which approach is superior in terms of supporting embryo development remains inconclusive (Sfontouris *et al.*, 2016; Werner *et al.*, 2016).

An additional layer of complexity arises with the introduction of TLM, which raises the questions of whether it can detect subtle differences in embryo development between sequential and single-step media. One of the first study to explore this was conducted by Ciray *et al.* (2012), who performed a randomized study on 446 oocytes. These oocytes were divided into two groups, culture in either single step or sequential media produced by the same manufacturer, and cultured in the same time-lapse incubator. The study found that embryos cultured in single-step media exhibited earlier fading of the PN and earlier cleavage up to the five-cell stage compared to those cultured in sequential media. Furthermore, in embryo that implanted, the time t2 to t4 were significantly shorter when cultured in single-step media. However, the clinical pregnancy rates did not differ significantly between the two groups (Ciray *et al.*, 2012). A similar finding was reported by Kazdar *et al.* (2017), who also observed no significant differences in clinical outcomes between embryos cultured in single-step and sequential media. On the other hand, other studies have failed to identify any notable morphokinetic differences between the two approaches (Basile *et al.*, 2013; Sfontouris *et al.*, 2017). As a result, current data have not shown a clear advantage of either single-step or sequential media in terms of clinical pregnancies outcomes, whether embryos are cultured in standard incubators or using TLM.

As previously mentioned, TLM offers the advantage of preventing embryos from exposure to environmental fluctuations, including changes in pH, temperature, and gases concentrations (CO_2_ and O_2_), thus more closely emulating the conditions found in vivo. One of the key factors influencing embryo development is the concentration of oxygen, and an ongoing debate persists regarding culturing embryos at low oxygen levels versus ambient levels (Sciorio & Smith, 2019). The oxygen concentration within the mammalian female reproductive tract is between 2% and 8% (Fischer & Bavister, 1993). When embryos are exposed to atmospheric oxygen levels, there is a risk of increased production of reactive oxygen species, which can disrupt normal embryo metabolism and gene expression (Fischer & Bavister, 1993; Rinaudo *et al.*, 2006; Wale & Gardner, 2012). There is considerable evidence suggesting that culturing embryo in lower oxygen concentration, such as 5% improves pregnancy outcomes. Studies have shown that embryos cultured in 5% oxygen display better developmental progression and higher implantation rates (Meintjes *et al.*, 2009; Bontekoe *et al.*, 2012). A large-scale prospective randomized multicentre study involving 1,563 oocytes confirmed that adding antioxidants to the culture media significantly improves embryo viability, implantation rates, and ongoing pregnancy rates. This improvement is likely due to the reduction of oxidative stress, which is harmful to developing embryos (Gardner *et al.*, 2020).

Furthermore, TLM helps mitigate the effects of environmental stressors, allowing for stable culture conditions that more closely resemble the in vivo environment. The control of oxygen concentration and the use of antioxidants have proven beneficial in reducing oxidative stress, thus enhancing embryo development and improving clinical outcomes. As research continues, the use of TLM and optimized culture conditions promises to refine embryo selection and improve the chances of successful pregnancies in ART cycles.

## EVOLUTION OF ARTIFICIAL INTELLIGENCE (AI) AND TLM

Although TLM has been proposed since the 1929 (Lewis & Gregory, 1929), the technology became commercially available only about a decade ago. In comparison to other advancements in cell biology, TLM can still be considered in its early stages, with much room for improvement. Looking ahead, it is expected that developments related to image collection will continue. The integration of fluorescence and confocal microscopy with TLM, allowing for the morphokinetic observation of organelles and chromosomes, has been already proposed (Holubcová *et al.*, 2015; Patel *et al.*, 2015), along with fluorescence live-cell imaging of human embryos (Hashimoto *et al.*, 2016).

However, one of the challenges of TLM is the difficulty assessing and interpreting the vast amount of data it generates. This challenge presents an opportunity for the evolution of artificial intelligence (AI) and the use of more powered computer to analyse the large volume of image, with the goal of identifying specific parameters that correlate with embryo viability and pregnancy outcomes. In that context, software programs are being developed as automatic alternatives to standardize time-lapse annotations (Yeung *et al.*, 2018). Unlike in other medical fields, ART has yet to fully explore the advantages of AI for automated embryo evaluation and selection.

It has been hypothesized that an AI system trained on thousands of embryo images and videos would later be able to identify and predict embryo quality autonomously. This could help reduce human error, standardize annotations, and allow embryologists to focus on other critical tasks. A study conducted by Khosravi *et al.* (2019) used AI in conjunction with TLM to analyse clinical data from 2,182 embryos and about 50,000 images. Their model achieved an area under the curve (AUC) of *>*0.98 when predicting BL quality (Khosravi *et al.*, 2019). In another retrospective study, a deep learning approach has applied to automatically annotate 10,638 embryos videos from eight different IVF units across four countries. The results demonstrated that the deep learning model could predict foetal heartbeat and pregnancy outcomes from TLM videos with an AUC of 0.93 (Tran *et al.*, 2019). Although these studies were retrospective, they highlight the promising potential of AI in predicting embryo viability and implantation success.

However, further prospective randomized controlled trials are required to evaluate the clinical significance of AI in IVF (Khosravi *et al.*, 2019; Tran *et al.*, 2019). Before AI based approaches can be clinically implemented, they will need to undergo rigours validation to ensure their reliability and accuracy.

## AI: EMBRYO ASSESSMENT AND AUTOMATIC ANNOTATION

Imaging is one of the most prominent applications of AI, particularly in the field of human embryology. AI has proven effective in object identification, shape prediction, and texture analysis, with successful applications in other medical fields. In clinical embryology, AI can be applied to automatic embryo annotation, embryo grading, and embryo selection for implantation. These advancements have the potential to standardize and optimize embryo evaluation, especially with the integration TLM, which enables precise tracking of cellular divisions and detection of both normal and abnormal embryo development. AI applications in TLM can revitalize this technology and improve embryo assessment.

Commercial TLM systems, such as Geri, and ESCO, claim to incorporate machine learning (ML) into their evaluation software, although the manufacturers have not disclosed platform details or performance accuracy. Currently, embryo annotation during TLM incubation is a manual task performed by embryologists, who must annotate every features. However, this process is subject to high intraand inter-operator variability. As a result, AI-based automatic annotation systems with high accuracy and reliability are essential to reduce variability and standardize embryo assessment. The EmbryoScope by Vitrolife using AI technology, has developed an algorithm called iDAScore, that automatically analyses full time-lapse sequences to support embryo evaluation and identify embryos with the highest likelihood of implantation within a cohort. Recent studies have demonstrated the success of automatic, non-human-mediated embryo annotation systems (Dirvanauskas *et al.*, 2019; Raudonis *et al.*, 2019; Feyeux *et al.*, 2020; Zaninovic & Rosenwaks, 2020). These systems must meet specific criteria: they should be fast, accurate, reproducible, and capable of distinguishing between normal and abnormal developmental features. Furthermore, these systems need to detect morphological features such as uneven size, vacuoles, and granularity, along with nuclear abnormalities like multinuclear blastomeres. The ideal system would assign appropriate weight to each characteristic for accurate prediction.

To achieve automatic annotation, several image-processing techniques, such as cell shape extraction, segmentation, can be integrated with AI, typically using convolutional neural networks (CNNs). One challenge is to recognize and detect embryos within the culture well and create an automatic region of interest (ROI). This can be accomplished using cascade classifiers or segmentation. Research has shown that the Inception V3 CNN model can perform automatic cell annotation without preprocessing the ROI, achieving high accuracy (93.9%) on human TLM images up to the eight-cell stage. Recent studies, such as those by Feyeux *et al.* (2020), have successfully used segmentation tools to automatically annotate embryos up to the BL stage These systems rely on quantifying features like zona pellucida thickness to identify BL expansion.

The automated tool was validated with annotations from embryologists, demonstrating the effectiveness of these automated tools and highlighting the potential of AI to improve embryo evaluation in ART (Feyeux *et al.*, 2020).

## AI AND EMBRYO GRADING

Advancements in AI within embryology have primarily focused on embryo grading, particularly at the blastocyst stage, as this is strongly correlated with implantation success. Despite the widespread use of the Gardner grading system (Gardner & Schoolcraft, 1999), its combination of numerical and letter grades often leads to inconsistencies. To address this, a simplified numerical BL score has been proposed, incorporating quantitative features of the blastocyst, such as expansion, ICM and TE (Zhan *et al.*, 2020). However, grading variability persists even with the use of TLM. A study of ten embryologists revealed significant grading differences, while AI algorithms demonstrated superior performance in embryo evaluation and selection (Bormann *et al.*, 2020).

The main challenge in automating this process lies in ensuring the availability of high-quality training data, as AI learns from images that have been manually graded by embryologists. Ideally, ML systems should function autonomously, without human intervention. Early attempts to automate BL grading involved differentiating the ICM from the TE through image segmentation, using support vector machines applied to two-dimensional images. AI applications have also extended to animal research. For example, a study by Matos *et al.* (2014) demonstrated that automatic morphological classification of mouse blastocysts could achieve 95% accuracy. The same research group applied an AI tool to successfully assess bovine embryos (Rocha *et al.*, 2017), which was a critical step for applying AI methods to human embryo quality evaluation and prediction (Saeedi *et al.*, 2017). For instance, CNN-based systems have been developed to automatically grade BL features, including ICM and TE morphology.

One approach, using TLM data, involved preprocessing images with cropping and applying a CNN combined with recurrent neural networks. This method predicted BL components with high accuracy of 97.8% for ICM and 98.1% for TE (Kragh *et al.*, 2019). Another method, which used static images, achieved 96% accuracy in predicting BL expansion, 91% for ICM, and 84% for TE (Chen *et al.*, 2019). The debate between using static images versus videos for embryo assessment continues. Videos, providing dynamic data, may offer a more comprehensive view of embryo development, while static images capture critical time points, such as 66 or 110 hours post-ICSI, that can accurately assess developmental competence. However, static images are limited in evaluating dynamic events, like blastocoel collapse, which could interfere with assessment. These approaches require further testing on similar datasets to determine which method is more effective for embryo selection (Manna *et al.*, 2013). Recent work by Khosravi *et al.* (2019) suggests that AI can be clinically applied for embryo classification. Their study, focusing on blastocysts at 110 hours post-ICSI, achieved over 98% accuracy in distinguishing high grade from low grade embryos. An innovative approach involves using smartphone-based systems to evaluate embryos, with AI algorithms trained on high-quality images and applied to lower-quality images captured by portable devices. This approach achieved around 90% accuracy in distinguishing blastocysts from non-blastocysts, demonstrating AI adaptability and potential for clinical use in diverse settings (Kanakasabapathy *et al.*, 2019).

Several AI startups are now developing algorithms to predict embryo development and implantation across various stages. Despite claims of high predictive accuracy, these AI platforms are still evolving, with CNNs proving particularly effective for image analysis. However, many studies have been based on limited training data, raising concerns about the generalizability of AI models. A significant challenge in ML is the use of unbalanced datasets, which can skew predictions towards the majority class, highlighting the need for larger and more diverse data sets for training.

## EMBRYO SELECTION TO IMPROVE IMPLANTATION

Embryo selection in ART represents a critical aspect of improving implantation and pregnancy success. Traditional methods rely on morphological evaluation, but this technique suffers from significant variability and inconsistency between operators. AI offers a promising solution, aiming to improve embryo selection and predict implantation potential more objectively. However, AI models focused on predicting live birth rates, may overlook other essential factors, such as uterine conditions, which also influence pregnancy success.

Several statistical models have been developed to predict implantation success by analysing morphokinetic embryos parameters using TLM. These parameters are linked to embryo quality and can provide valuable insights into implantation potential. Some AI-based systems use TLM data to predict implantation at both the cleavage and blastocyst stages. For instance, a deep learning model developed by Tran *et al.* (2019) aimed to predict foetal heart rate by analysing TLM videos, claiming to offer a fully automated system for embryo assessment. While the study used a large dataset, concerns have been raised about the inclusion of abnormal or discarded embryos, which could lead to unbalanced training data and skew the results. Additional study (Chavez-Badiola *et al.*, 2020), which used a relatively small size of approximately 200 transferred blastocysts from two clinics, applied static images to predict implantation rate and achieved an accuracy of about 70%. However, the predictive value of the model remained unclear, especially when compared to traditional grading methods. A further multicenter study using a large retrospective dataset tested an AI platform for predicting implantation rate using single blastocyst images (VerMilyea *et al.*, 2020). The model showed an accuracy of 67.7%, with sensitivity (true positive) of 71.1% and specificity (true negative) of 65.3%. The model’s predictions were compared to those made by embryologists across eleven different IVF units, resulting in an overall improvement of 30.8%. However, the study’s methodology for grading embryos and the variation in grading practices across laboratories could influence the results.

Despite the promise of AI, the question remains whether a universal AI system for embryo evaluation and selection will be effective across different IVF laboratories. Local conditions, such as culture media, incubators, and assessment methods, could significantly impact data interpretation. To improve the generalizability of AI systems, it is essential to use diverse datasets from multiple labs during training. Additionally, incorporating other clinical parameters, such as patient age, ovarian reserve, and stimulation protocols, is necessary to enhance AI’s predictive accuracy. Finally, while AI offers significant potential in embryo selection, more research is needed to address challenges like data quality, model generalizability, and the integration of multiple clinical factors (Zaninovic *et al.*, 2019). A comprehensive AI model incorporating both embryo imaging and morphokinetics, along with clinical data, could ultimately lead to better outcomes in ART cycles.

## POTENTIAL CORRELATION BETWEEN TLM AND EMBRYOS ANEUPLOIDY

Aneuploidy refers to the presence of an incorrect number of chromosomes in a cell, such as 45 or 47 chromosomes instead of the normal 46. Aneuploidy is a significant concern in *in-vitro* human embryos obtained through ART treatments. The transfer of aneuploid embryos may result in implantation failure, miscarriages, or the birth of offspring with a range of potential abnormalities (Sciorio & Dattilo, 2020). The conventional procedure to investigate aneuploidy in human embryos is called preimplantation genetic testing for aneuploidy (PGT-A), previously known as preimplantation genetic screening (PGS). This procedure involves an IVF cycle in which embryos are biopsied and screened for chromosomal abnormalities before being transferred to the uterus. The procedure was first introduced by Handyside *et al.* (1990). However, PGT-A is an expensive technology, is not permitted in some countries, and there is ongoing debate regarding its cost-effectiveness, the invasiveness of the procedure, and its clinical efficiency (Sermon *et al.*, 2016; Sciorio & Dattilo, 2020).

It has been hypothesized that TLM could be used to identify embryo aneuploidy, offering a cheaper, faster, and less invasive evaluation approach. Several studies have correlated morphokinetic parameters observed through TLM with the likelihood of selecting chromosomally normal embryos. It has been suggested that cell division timing need to be within an optimum range to ensure the proper execution of cellular processes before cytokinesis (Davies *et al.*, 2012; Campbell *et al.*, 2013a; 2013b; Montag, 2013; Swain, 2013; Chawla *et al.*, 2015). Davies *et al.* (2012) found that aneuploid embryos exhibited delays in the first two cleavages and prolonged transitions between 2-cell and 4-cell stages. The authors also observed that abnormal embryos had higher rates of irregular divisions and asynchronous PN disappearance compared to normal embryos. Chavez *et al.* (2012) investigated the relationship between genetic status and morphokinetic parameters, demonstrating that euploid embryos have distinct timing for the first cell divisions up to the 4-cell stage. Chawla *et al.* (2015) assessed several morphokinetic features, including the timing of second polar body extrusion, pronuclei appearance and fading, and the duration of first, second, and third cleavages in 460 embryos to distinguish abnormal embryos. The results revealed significant differences in morphokinetic parameters between euploid and aneuploidy embryos (Chawla *et al.*, 2015). Campbell *et al.* (2013a; 2013b) used TLM to develop a model for identifying embryo aneuploidies. They found the timing of early blastulation and full blastocyst formation as a key features related to embryo euploidy. Basile *et al.* (2014) investigated the differences in cleavage timing between chromosomally normal and abnormal embryos to help identify normal embryos. They found that normal and abnormal embryos exhibited different kinetic behaviours and proposed an algorithm as a non-invasive tool to improve the likelihood of selecting genetically normal embryos (Basile *et al.*, 2014). A comprehensive review on the use of TLM to identify and select euploid embryos was recently published by Reignier *et al.* (2018). They concluded that, although several studies demonstrated significant differences in morphokinetic parameters between euploid and aneuploid embryos, none provided adequate evidence to recommend the clinical use of TLM for identifying embryo aneuploidies. Consequently, the use of time-lapse technology should not be considered as a replacement for PGT-A (Reignier *et al.*, 2018).

## CONCLUSIVE REMARKS

Despite significant progress in ART across the glove, many IVF clinics still rely on standard morphological evaluation for embryo selection, a method that has inherent limitations. To improve the selection process, it is crucial to integrate novel, objective criteria that can better predict which embryos should be selected for transfer in IVF cycles. In that context, the introduction of TLM offers exciting new morphokinetic features throughout *in-vitro* culture. This technology provides embryologists with deeper insights into key developmental stages of embryos, ultimately enhancing the embryo selection process and improving the chances of a successful pregnancy. Detection of atypical embryo phenotypes has proven to be essential for eliminating embryos with poor prognoses, which could lead to unsuccessful pregnancies. With the current advancements in TLM technology, a continuous and stable embryo culture environment can be maintained, allowing embryologists to detect previously unnoticed or undetectable aspects of embryonic development. These include abnormal events like direct cleavage into three cells, which have been shown to negatively impact clinical pregnancy outcomes. Looking forward, with the further advancement of AI over the next decade, it is expected that TLM will become a well-established and indispensable tool for embryo selection. By integrating TLM with non-invasive analytical methods, it is likely to be routinely utilized in IVF clinics. As a result, TLM and AI will become a vital part of the toolkit for embryologists, ensuring better embryo culture conditions and more successful outcomes in ART treatments.
